# Isotopic signatures reveal zinc cycling in the natural habitat of hyperaccumulator *Dichapetalum gelonioides* subspecies from Malaysian Borneo

**DOI:** 10.1186/s12870-021-03190-4

**Published:** 2021-09-27

**Authors:** Antony van der Ent, Philip Nti Nkrumah, Mark G. M. Aarts, Alan J. M. Baker, Fien Degryse, Chris Wawryk, Jason K. Kirby

**Affiliations:** 1 Centre for Mined Land Rehabilitation, Sustainable Minerals Institute, The University of Queensland, Queensland 4072 St Lucia, Australia; 2 Laboratoire Sols et Environnement, Université de Lorraine-INRAE, UMR 1120, Nancy, France; 3 Laboratory of Genetics, Wageningen University and Research, Wageningen, The Netherlands; 4 School of BioSciences, The University of Melbourne, Victoria Melbourne, Australia; 5 Soil Sciences, University of Adelaide, South Australia Adelaide, Australia; 6 Industry Environments Program, CSIRO Land and Water, Environmental Assessment and Technologies, Adelaide, South Australia Australia

**Keywords:** Cycling, Hyperaccumulation, Phloem, Southeast Asia, Weathering, Zinc isotopes

## Abstract

**Background:**

Some subspecies of *Dichapetalum gelonioides* are the only tropical woody zinc (Zn)-hyperaccumulator plants described so far and the first Zn hyperaccumulators identified to occur exclusively on non-Zn enriched 'normal' soils. The aim of this study was to investigate Zn cycling in the parent rock-soil-plant interface in the native habitats of hyperaccumulating *Dichapetalum gelonioides* subspecies (subsp. *pilosum* and subsp. *sumatranum*). We measured the Zn isotope ratios (δ^66^Zn) of *Dichapetalum* plant material, and associated soil and parent rock materials collected from Sabah (Malaysian Borneo).

**Results:**

We found enrichment in heavy Zn isotopes in the topsoil (δ^66^Zn 0.13 ‰) relative to deep soil (δ^66^Zn -0.15 ‰) and bedrock (δ^66^Zn -0.90 ‰). This finding suggests that both weathering and organic matter influenced the Zn isotope pattern in the soil-plant system, with leaf litter cycling contributing significantly to enriched heavier Zn in topsoil. Within the plant, the roots were enriched in heavy Zn isotopes (δ^66^Zn ~ 0.60 ‰) compared to mature leaves (δ^66^Zn ~ 0.30 ‰), which suggests highly expressed membrane transporters in these *Dichapetalum* subspecies preferentially transporting lighter Zn isotopes during root-to-shoot translocation. The shoots, mature leaves and phloem tissues were enriched in heavy Zn isotopes (δ^66^Zn 0.34**–**0.70 ‰) relative to young leaves (δ^66^Zn 0.25 ‰). Thisindicates that phloem sources are enriched in heavy Zn isotopes relative to phloem sinks, likely because of apoplastic retention and compartmentalization in the *Dichapetalum* subspecies.

**Conclusions:**

The findings of this study reveal Zn cycling in the rock-soil-plant continuum within the natural habitat of Zn hyperaccumulating subspecies of *Dichapetalum gelonioides* from Malaysian Borneo. This study broadens our understanding of the role of a tropical woody Zn hyperaccumulator plant in local Zn cycling, and highlights the important role of leaf litter recycling in the topsoil Zn budget. Within the plant, phloem plays key role in Zn accumulation and redistribution during growth and development. This study provides an improved understanding of the fate and behaviour of Zn in hyperaccumulator soil-plant systems, and these insights may be applied in the biofortification of crops with Zn.

**Supplementary Information:**

The online version contains supplementary material available at 10.1186/s12870-021-03190-4.

## Background

Metal hyperaccumulator plants have the ability to accumulate high concentrations of potentially toxic trace elements in their living shoots without suffering any toxicity symptom (Baker and Brooks 1989; Reeves 2003; Reeves et al. 2018). For zinc (Zn), the notional hyperaccumulation threshold is set at 3000 µg g^− 1^ (Krämer et al. 2007; van der Ent et al. 2013). This contrasts with Zn concentrations in most non-hyperaccumulator plant species that are typically between 30 and 100 µg g^− 1^ (Nouvas et al. 2018). Zinc hyperaccumulator plants are a minority (~ 30 species) of the ~ 700 hyperaccumulator plant species currently documented globally (Reeves et al. 2018). All Zn hyperaccumulator species that have been identified to date are herbaceous plants, except for some subspecies of *Dichapetalum gelonioides*, which is a (tropical) woody species (Nkrumah et al. 2018). All herbaceous Zn hyperaccumulators only hyperaccumulate Zn when growing in Zn-rich metalliferous soils, with the notable exception of *Arabidopsis halleri* and several *Noccaea* species (notably *N. caerulescens*) which occur on both Zn-rich and non-contaminated soils and hyperaccumulate on both. The foliar tissues of *A. halleri* and *N. caerulescens* can accumulate 53,900 and 12,300 µg Zn g^− 1^, respectively, when growing on uncontaminated 'normal' soils with < 350 µg Zn g^− 1^ (Tang et al. 2012; Stein et al. 2017). *Arabidopsis halleri and N. caerulescens* are the two key model species for the study of Zn hyperaccumulation and their ecology, physiology, molecular biology and genetics have broadened our understanding of metal regulation in plants (Bert et al. 2000; Assunção et al. 2003; Schwartz et al. 2003; Lin et al. 2014; Stein et al. 2017; Ricachenevsky et al. 2021).

Zinc hyperaccumulator plants take up Zn from the same Zn labile soil pools as non-hyperaccumulator plants, but at higher rates (Deng et al. 2014). Zinc transport within plants occurs by means of several families of metal transporters, and the hyperaccumulation trait likely evolved from mechanisms involved in the regulation of Zn homeostasis (Ajeesh Krishna et al. 2017; Corso and García de la Torre 2020; Hanikenne and Nouet 2011; Manara et al. 2020). The Zn hyperaccumulation trait is correlated with high and constitutive expression of genes normally involved in the Zn deficiency response, such as in metal transport, in the biosynthesis of metal chelators and in cellular defences to oxidative stresses (Andresen et al. 2018; Assunção et al. 2001; Hammond et al. 2006; van de Mortel et al. 2006; Talke et al. 2006; Hanikenne and Nouet 2011). The current hypothesis is evolution of Zn hyperaccumulation likely resulted from an increased uptake of Zn by roots (Cappa and Pilon-Smits 2014; Hanikenne and Nouet 2011; Merlot et al. 2021). These plants then translocate the Zn to the shoots and sequester it in foliar cells, which provide a selective advantage through protection against herbivory (Boyd 2012; Boyd and Martens 1994; Cabot et al. 2019; Pollard and Baker 1997; Stolpe et al. 2017). Thereafter, in subsequent cycles of selection, hyperaccumulation evolved to become more efficient by improving Zn translocation and shoot sequestration or detoxification (Assunção et al. 2001; Lin et al. 2014; Schvartzman et al. 2018).

The mechanisms underlying Zn hyperaccumulation are still far from being fully understood. In addition, the role of Zn hyperaccumulator plants in Zn biogeochemical cycling has remained relatively unexplored. However, complementing elemental analysis with stable Zn isotope ratios can help improve our understanding of Zn cycling in the rock-soil-plant interface. Organic matter cycling and chemical weathering can lead to Zn isotope fractionation (Opfergelt et al. 2017; Viers et al. 2015). Zinc isotope signatures in plants is indicative of the main translocation and storage pathways (Moynier et al. 2009; Smolders et al. 2013; Wiggenhauser et al. 2018). Notably, Zn isotope fractionation between shoot organs varies between plant species (Caldelas and Weiss 2017; Wiggenhauser et al. 2018). In plants from a pristine tropical watershed, preferential translocation of depleted heavy Zn isotopes into adjacent cells of xylem vessels led to lighter Zn isotope ratio enrichment in leaves of trees relative to stems (Viers et al. 2007). However, in reed canary grass, light Zn isotopes preferentially accumulated in stems compared to heavy Zn isotopes in the leaves (Aucour et al. 2015). Moreover, young leaves were found to have a lighter Zn isotopic signature compared to mature leaves of reed (Caldelas et al. 2011). Sorption to cell walls, xylem unloading, and storage in vacuoles by binding Zn to organic acids are suggested for the enrichment of heavy Zn isotopes in mature leaves relative to stems in reed (Aucour et al. 2015). In wheat, preferential enrichment of light Zn isotopes accumulate in phloem sinks, whereas phloem sources are enriched in heavy Zn isotopes, likely because of apoplastic retention and compartmentalization (Wiggenhauser et al. 2018). *Noccaea caerulescens*, growing on a granite non-contaminated site, preferentially incorporates light Zn isotopes into the leaves (δ^66^Zn -0.10–0.03 ‰) relative t the o roots (δ^66^Zn 0.43–0.49 ‰) (Tang et al. 2012). Similarly, *A*. *halleri* preferentially incorporates lighter Zn isotopes in its shoots relative to the roots (Aucour et al. 2015).

*Dichapetalum* is a large genus comprising more than 150 species primarily occuring in tropical and subtropical regions, with most species known from Africa (Punt 1975). *Dichapetalum gelonioides* is a woody shrub or small tree which occurs in Southeast Asia. Four subspecies are known to date in Sabah Malaysian Borneo): subsp. *gelonioides*, subsp. *pilosum* subsp. *sumatranum* and subsp. *tuberculatum*. For detailed information on their habitats and main taxonomic characteristics, see Nkrumah et al. (2018). These *D. gelonioides* subspecies differ in their metal accumulation characteristics: *D. gelonioides* subsp. *tuberculatum* is a strong nickel (Ni) hyperaccumulator when growing on ultramafic soils, resulting in > 26,000 µg g^− 1^ dry mass foliar Ni, and a strong Zn hyperaccumulator when growing on non-contaminated soils, leading to up to 30,000 µg g^− 1^ dry mass foliar Zn (Baker et al. 1992). *Dichapetalum gelonioides* subsp. *sumatranum* and *D. gelonioides* subsp. *pilosum* are strong Zn hyperaccumulators on non-contaminated soils, reaching up to 15,700 µg g^− 1^ and 26,400 µg g^− 1^ dry mass foliar Zn respectively (Baker et al. 1992; Nkrumah et al. 2018). *Dichapetalum gelonioides* subsp. *gelonioides* is a non-accumulator. The ability of *D. gelonioides* to hyperaccumulate Zn from soils with only background concentrations of Zn (< 100 µg g^− 1^) is unique, and contrary to other identified Zn hyperaccumulators, except for the model species *A. halleri* and *N. caerulescens*. It is also the sole Zn hyperaccumulator species known to exclusively occur on non-Zn metalliferous soils, and the only (humid) tropical and woody Zn-hyperaccumulator plant species described so far (van der Ent et al. 2018; Nkrumah et al. 2018).

Hyperaccumulator plants can play a substantial role in the cycling of elements in their local ecosystems, especially in the upper soil horizons, through elemental uptake in the roots, litterfall and litter recycling/biodegradation (Echevarria 2021; Ratié et al. 2019). In Mediterranean climatic regions, local Ni hyperaccumulator plants directly influence the composition of available and total Ni pools in the ultramafic soils (Estrade et al. 2015). The authors suggested that Ni hyperaccumulators play a major role in maintaining high Ni concentrations in the surface soils during weathering and pedogenesis. However, to-date, the role of plants in elemental cycling are not yet fully understood (Zelano et al. 2020). Little is known about the Zn cycling in the parent rock-soil-Zn hyperaccumulator plant interface in tropical regions. This data could provide information about pathways and processes of Zn that control Zn recycling in the habitats of tropical Zn hyperaccumulator plants. Therefore, the primary aim of this study was to use Zn isotope ratios to investigate Zn cycling in the parent rock-soil-plant interface in the habitats of the Zn hyperaccumulators *D.* subsp. *pilosum* and subsp. *sumatranum*.

## Results

### Zinc concentrations in the ***Dichapetalum*** plant tissues, soils and bedrock samples

**Fig. 1:**
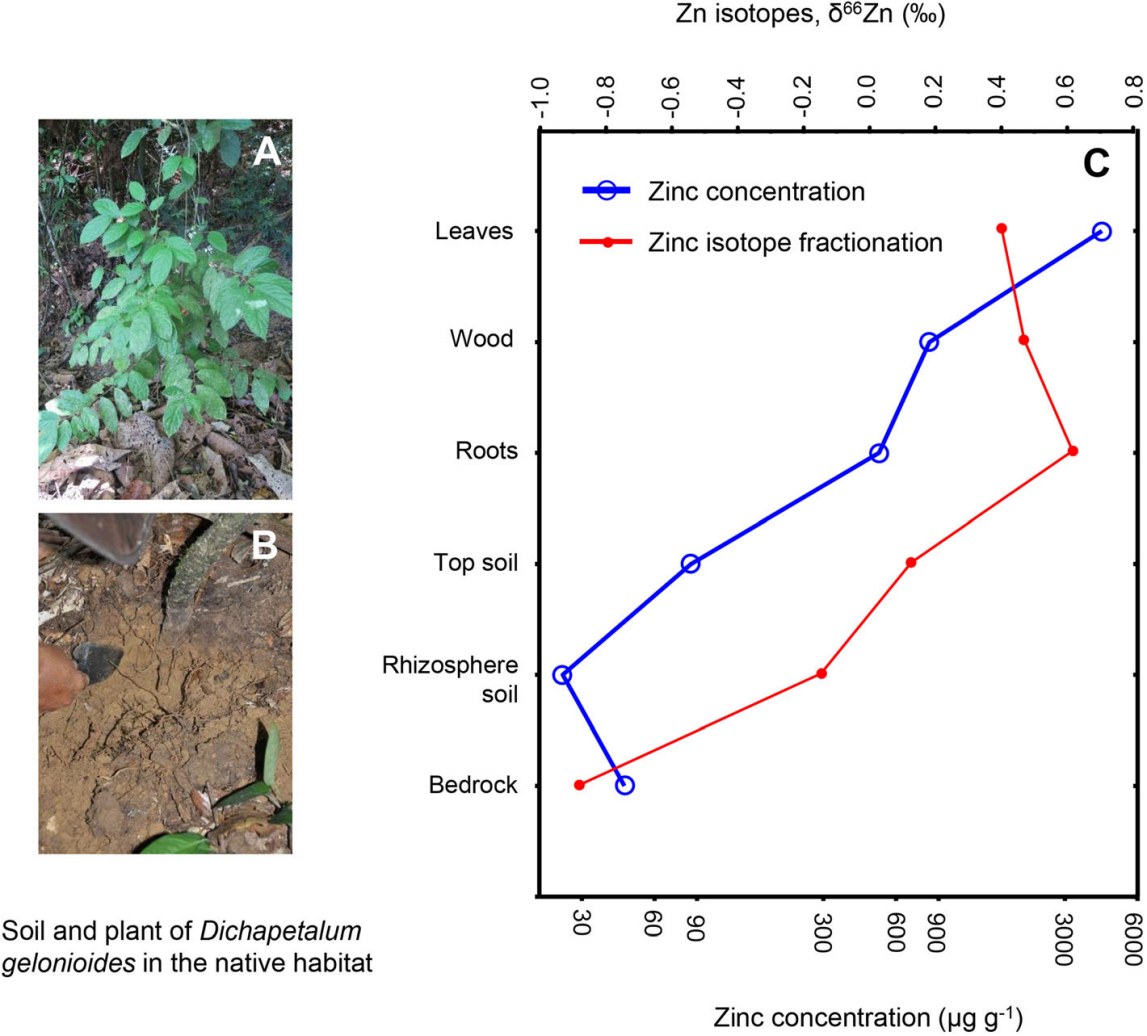
(**A**) Whole plant of *Dichapetalum gelonioides* subsp. *pilosum* in the native habitat in Malaysia. (**B**) Soil profile at the base of this *Dichapetalum* individual. (**C**) Mean Zn concentrations (µg g^−1^) (n = 4) (in blue) and δ^66^Zn isotope ratios (‰) (n = 2) (in red) of *D.*subsp. *pilosum* plant tissues and corresponding topsoil (0–2 cm depth), rhizosphere soil (at 10–30 cm depth) and bedrock (at > 30 cm depth). The plant tissues include roots, wood and leaves

The Zn concentrations in the soil and bedrock samples collected from the native habitats of *D.* subsp. *pilosum* were < 100 µg g^− 1^ dry mass (Fig. 1; Table 1 and Supplementary Table 1). The potentially phytoavailable Zn concentrations (diethylene triamine pentaacetic acid (DTPA)-extractable Zn) were enriched in the topsoil (0–2 cm), reaching 30 µg g^− 1^ dry mass, whereas that in the rhizosphere soils (0.85–5.5 µg g^− 1^ dry mass), the B-horizon and bedrock (< 1 µg g^− 1^ dry mass) were relatively depleted. The strontium nitrate (Sr(NO_3_)_2_)-extractable Zn concentrations followed a similar trend, but the rhizosphere soils had higher concentrations relative to the topsoil. The topsoil and the B-horizon of *D.* subsp. *pilosum* had circum-neutral soil pH, whereas the rhizosphere soils of *D.* subsp. *pilosum* and *D.* subsp. *sumatranum* were strongly acidic (Table 1 and Supplementary Table 1).

**Table. 1:** Soil pH, strontium nitrate (Sr(NO_3_)_2_)- and diethylene triamine pentaacetic acid (DTPA)-extractable Zn concentrations and total Zn concentrations of rhizosphere soils collected from the native habitat of *D.* subsp. *pilosum* and *D.* subsp. *sumatranum*. The number of samples is 4 for each subspecies; pH and concentrations are given as ranges and means

Parameter	*Dichapetalum gelonioides* subsp. *pilosum*	*Dichapetalum gelonioides* subsp. *sumatranum*
**pH**	4.35–4.95 [4.70]	4.80–4.95 [4.85]
**Sr(NO**_**3**_ **)**_**2**_ **-Zn (µg g** ^**− 1**^ **)**	0.05–4.0 [1.5]	0.65–6.0 [3.0]
**DTPA-Zn (µg g** ^**− 1**^ **)**	0.75–5.5 [2.05]	0.75–8.5 [4.5]
**Total Zn (µg g** ^**− 1**^ **)**	10–45 [25]	55–65 [60]

**Fig. 2:**
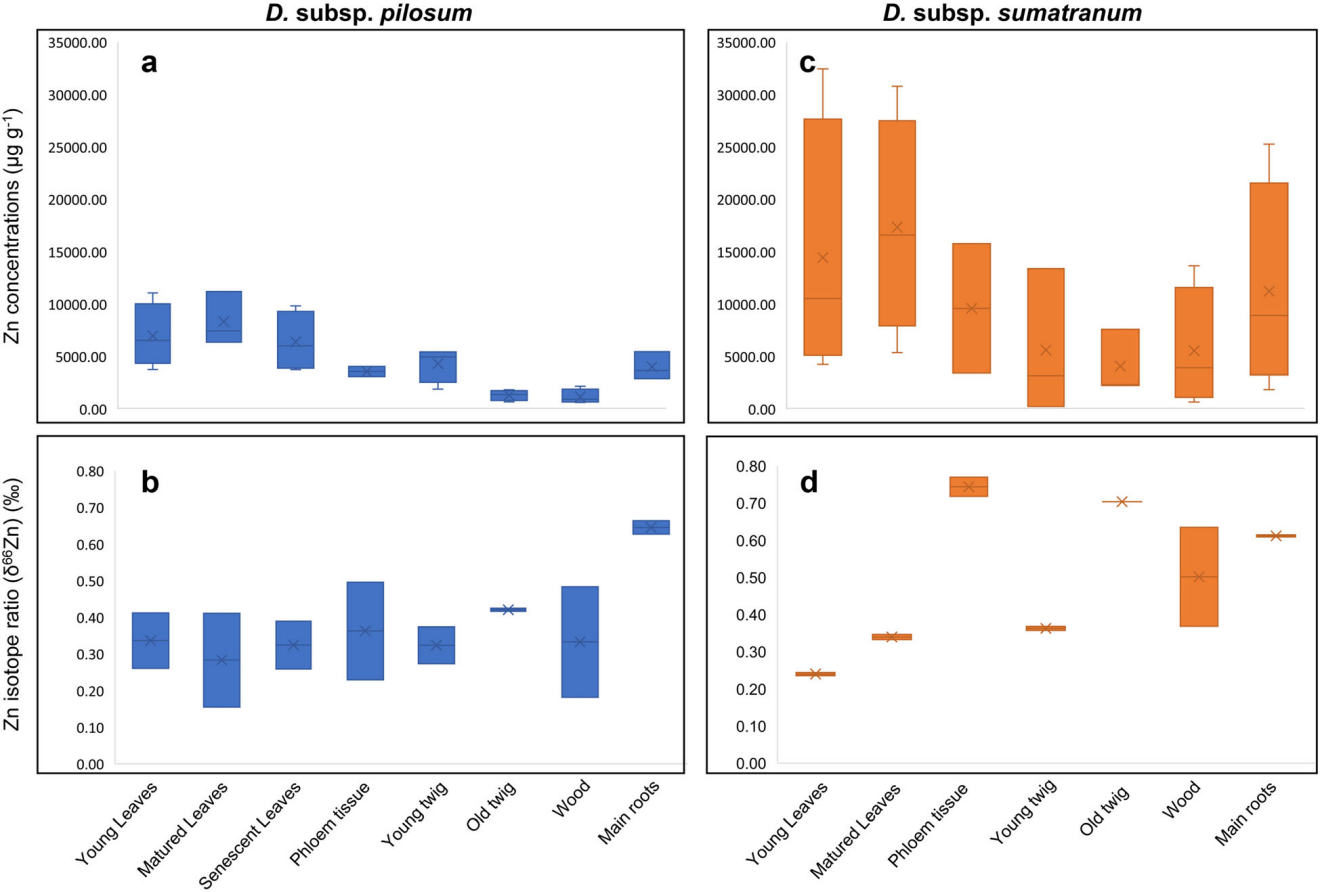
Zinc (Zn) concentrations (µg g^− 1^) in the various plant fractions of (**a**) *Dichapetalum* subsp. *pilosum* (in blue) and (**c**) *Dichapetalum* subsp. *sumatranum* (in orange) (n = 4), and the corresponding δ^66^Zn isotope ratios (‰) (n = 2) (**b**, **d**). One-way analysis of variance showed significant differences among the Zn concentrations in the various plant fractions of *Dichapetalum* subsp. *pilosum* (*p* < 0.05), whereas that in *Dichapetalum* subsp. *sumatranum* did not show significant differences (*p* > 0.05)

The Zn concentrations in the various plant tissues of *D.* subsp. *pilosum* and *D.* subsp. *sumatranum* were higher compared to the total and DTPA- and Sr(NO_3_)_2_-extractable Zn concentrations in the rhizosphere soils (Table 2). Both *D.* subsp. *pilosum* and *D.* subsp. *sumatranum* had similar Zn accumulation patterns in tissues, but *D.* subsp. *sumatranum* had relatively higher Zn concentrations (Table 2). The roots and leaves of *D.* subsp. *sumatranum* had Zn concentrations as high as 25,300 µg g^− 1^ and 32,400 µg g^− 1^, respectively, despite the comparatively lower Zn concentrations (total and extractable) in the rhizosphere soils (< 100 ug g^− 1^) (Figs. 1 and 2). The Zn concentrations in the senescent leaves, twigs and wood were relatively lower compared to the mature and young leaves, in both *Dichapetalum* subspecies (Fig. 2). Moreover, the mature leaves had relatively higher Zn concentrations (~ 1.2-fold) compared to the young leaves in both *Dichapetalum* subspecies. The phloem tissues of both *Dichapetalum* subspecies had relatively high Zn concentrations compared to the wood and twigs, reaching 15 800 µg g^− 1^ in *D.* subsp. *sumatranum* (Table 2).

**Table. 2:** Zinc concentrations (µg g^− 1^) (*n* = 4) and δ^66^Zn isotopic ratios (‰) (*n* = 2) for field collected plant samples from the native habitat of *D.* subsp. *pilosum* and *D.* subsp. *sumatranum*. Concentrations are given in ranges and mean, and isotope ratios are given in ranges

**Plant Fraction**	***Dichapetalum gelonioides*** ** subsp. ** ***pilosum***			***Dichapetalum gelonioides*** ** subsp. ** ***sumatranum***		
	**Zinc concentrations (µg g** ^**-1**^ **)**	**δ** ^**66**^ **Zn isotopic ratios (‰)**	**SD**	**Zinc concentrations (µg g** ^**-1**^ **)**	**δ** ^66^ **Zn isotopic ratios (‰)**	**SD**
**Young Leaves**	3750–11 100 [6970]	0.26–0.41	0.04–0.05	4250–32 400 [14 400]	0.24	0.06–0.08
**Old Leaves**	6370–11 200 [8350]	0.16–0.41	0.05–0.08	5370–30 800 [17 400]	0.33–0.35	0.04–0.07
**Senescent Leaves**	3740–9850 [6400]	0.26–0.39	0.04–0.07	–	–	–
**Phloem tissue**	3100–4050 [3580]	0.23–0.50	0.05	3420–15 800 [9610]	0.72–0.77	0.05–0.06
**Young twigs**	1910–5450 [4320]	0.27–0.37	0.06	250–13 400 [5620]	0.36–0.37	0.05
**Old twigs**	680–1850 [1330]	0.42	0.05–0.07	2250–7610 [4070]	0.7	0.07
**Wood**	640–2180 [1180]	0.18–0.48	0.03–0.06	780–13 700 [5550]	0.37–0.64	0.05
**Main roots**	2900–5470 [4010]	0.63–0.66	0.05–0.06	1860–25 300 [11 200]	0.61–0.62	0.04–0.05

*SD* standard deviation (instrumental analysis variability)

**Fig. 3:**
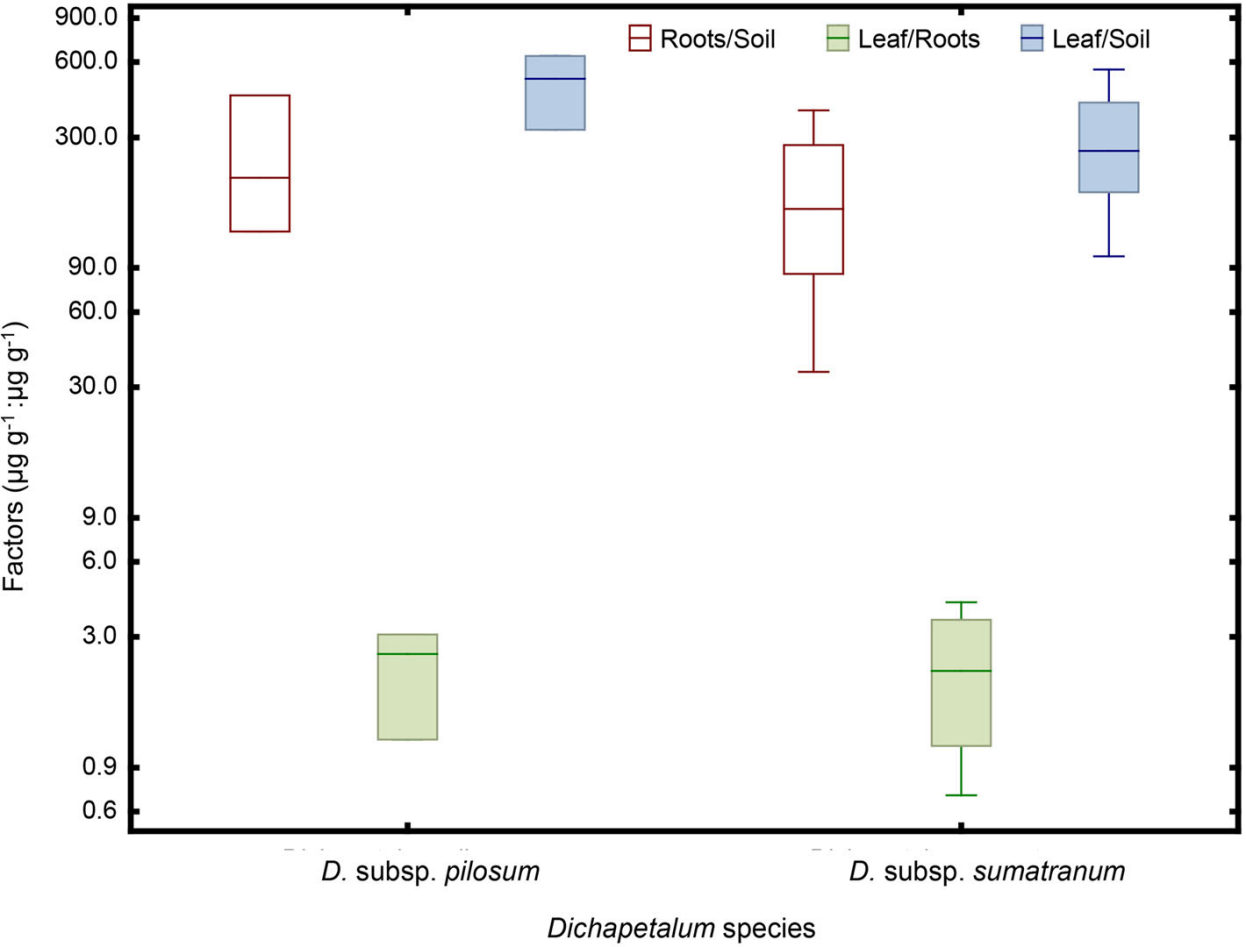
Zinc uptake (roots/soil) (in white), translocation (leaf/root) (in green) and bioaccumulation (leaf/soil) (in blue) factors of field collected *Dichapetalum* *gelonioides* subsp. *pilosum* and *Dichapetalum* *gelonioides* subsp. *sumatranum*. These factors are given in µg g^− 1^: µg g^− 1^. Key to symbols of boxplots: whiskers are ± standard deviation (SD)

*Dichapetalum* subsp. *pilosum* and *D.* subsp. *sumatranum* had a mean Zn uptake (root/soil) factor (UF) of 255 and 180, respectively (Fig. 3). Both *Dichapetalum* subspecies had a mean Zn translocation (leaves/root) factor > 1.0. The mean Zn Bioconcentration Coefficient (BC) (leaf/soil) for *D.* subsp. *pilosum* and *D.* subsp. *sumatranum* were 490 and 295, respectively (Fig. 3).

### Zinc isotopes in the soil profile and plant tissues

In the soil profile, the bedrock Zn was enriched in light Zn isotopes (δ^66^Zn -0.90 ‰) relative to the topsoil (δ^66^Zn 0.13 ‰) (Fig. 1). There was a heavy Zn isotope depletion along the soil profile (Fig. 1). The roots of both *D.* subsp. *pilosum* and *D.* subsp. *sumatranum* were enriched in heavy Zn isotopes (δ^66^Zn 0.61–0.66 ‰) relative to the mature leaves (δ^66^Zn 0.16–0.41 ‰) (Table 2). In the aerial parts of *D.* subsp. *sumatranum*, the mature leaves were preferentially enriched in heavy Zn isotopes (δ^66^Zn 0.33–0.35 ‰) relative to the young leaves (δ^66^Zn 0.25 ‰) (Table 2; Fig. 2). The phloem tissue was preferentially enriched in heavy Zn isotopes (δ^66^Zn 0.72–0.77 ‰) compared to all plant parts. Moreover, the young leaves Zn was preferentially enriched in light Zn isotopes compared to the old twigs and senescent leaves (Table 2). In *D.* subsp. *pilosum*, Zn in old twigs were preferentially enriched in heavy Zn isotopes (δ^66^Zn 0.42 ‰) relative to young twigs (δ^66^Zn 0.27–0.37 ‰).

## Discussion

Our findings reveal that the δ^66^Zn values of the soil profile in the native habitat of *Dichapetalum* subspecies decrease with soil depth, and the decrease is especially pronounced between the bedrock and soil, suggesting that both chemical weathering and organic matter decomposition in this habitat lead to Zn fractionation (Jouvin et al. 2009; Moynier et al. 2017; Opfergelt et al. 2017; Viers et al. 2015). We suggest that the contribution of organic matter to the Zn fractionation in the soil profile may be more than that of chemical weathering. In Icelandic soils derived from basalt, soil organic matter influences the Zn fractionation compared to mineral constituents (Opfergelt et al. 2017). In Central Siberia, chemical weathering of basaltic rocks does not lead to chemical and mineralogical differentiation down the soil profiles, resulting in minimal fractionation of Zn isotopes (Viers et al. 2015). The heavy isotope enrichment in the topsoil relative to deep soil and bedrock may be related to the decay of organic matter induced by microorganisms (Viers et al. 2015; Weiss et al. 2007). Humification processes lead to preferential enrichment of heavy Zn isotopes as complexation of Zn by high molecular weight organic compounds (i.e., humic and fulvic acids) favours the enrichment of heavy Zn isotopes (Jouvin et al. 2009). Our findings indicate that leaf litter recycling strongly influences the soil Zn budget, leading to Zn-enriched topsoil relative to deep soil and bedrock. In ultramafic ecosystems, vegetation composed of Ni hyperaccumulators can significantly influence Ni isotopic compositions (with enrichment in lighter Ni isotopes) through its remobilization in the upper soil horizons (Estrade et al. 2015; Paul et al. 2021; Zelano et al. 2020).

With regards to root-to-shoot translocation, our findings are consistent with previous studies showing preferential incorporation of light Zn isotopes in mature leaves relative to roots (Caldelas et al. 2011; Deng et al. 2014; Jouvin et al. 2012; Moynier et al. 2009; Tang et al. 2012, 2016; Viers et al. 2007; Wiggenhauser et al. 2018). In plant roots, there is preferential enrichment of heavy Zn isotopes in Zn retained by O-ligands of pectin-containing cell walls, particularly the Zn fraction that is tightly bound to such cell walls (Aucour et al. 2015; Tang et al. 2016). Hence, there is a preferential transfer of light Zn isotopes from roots to mature leaves through the xylem. Notably, O and N donor ligands preferentially bind heavy Zn isotopes (Fujii et al. 2014), which leads to an enrichment of light Zn isotopes in the cytosolic Zn^2+^ pool. Therefore, membrane transport likely favours light Zn isotopes (Caldelas and Weiss 2017). In the Zn hyperaccumulator *N. caerulescens*, the upregulation of heavy metal ATPases (HMAs) drives efficient root-shoot Zn translocation (Merlot et al. 2021; Papoyan and Kochian 2004; Schvartzman et al. 2018; Verbruggen et al. 2009), which likely leads to preferential transfer of light Zn isotopes to the xylem (Tang et al. 2012). Our results suggest that Zn transporters, like HMAs, may be highly expressed in the roots of Zn-hyperaccumulating subspecies of *D. gelonioides*, as observed in *N. caerulescens* (Tang et al. 2012; Merlot et al. 2021).

Zinc from mature leaves is redistributed *via* the phloem to young plant organs (Page and Feller 2015). The Zn concentrations in the young leaves of the *Dichapetalum* subspecies here are relatively high in comparison with other aerial parts. This implies that Zn redistribution by the phloem in *Dichapetalum* is highly effective, with both senescent and mature leaves supplying Zn to the young leaves *via* phloem redistribution. Zinc derived from the xylem (if the leaves are transpiring) replaces Zn released from the mature leaves, whereas the Zn from the senescent leaves is not replaced, leading to Zn depletion (Table 2). The Zn isotope ratios in the senescent and mature leaves compared to the young leaves suggest that light isotopes are preferably remobilized from the phloem sources. This is consistent with Zn transfer at flowering and full maturity in wheat where phloem sources (e.g., senescent straw) are preferentially enriched in heavy Zn isotopes, whereas phloem sinks (e.g., young leaves and grains) are enriched in light Zn isotopes (Wiggenhauser et al. 2018). Notably, the phloem tissues of *D.* subsp. *sumatranum* have the greatest heavy Zn isotopes enrichment (δ^66^Zn 0.72–0.77 ‰), whereas young leaves have the smallest (δ^66^Zn = 0.24 ‰) of the tissues examined. This finding suggests that Zn absorption to cell walls in the apoplastic space preferentially retains heavy isotopes in phloem sources (Aucour et al. 2015, 2017; Tang et al. 2016; Wiggenhauser et al. 2018). Moreover, these authors suggest Zn complexation and compartmentalization in cells contribute to the enrichment of light Zn isotopes in phloem sinks. Isotopically heavy Zn is preferentially sorbed to the cell walls of diatoms (John et al. 2007). The large enrichment of heavy Zn in the phloem tissue (a major phloem source) relative to the young leaves (phloem sink) of *D.* subsp. *sumatranum* also suggests that transporters and chelators within the phloem sap (Clemens 2019) preferentially complex/bind heavy Zn isotopes (Fujii et al. 2014).

## Conclusions

The findings from this study suggest that both weathering and Zn-enriched organic matter recycling influence the enrichment of heavy Zn isotopes in the topsoil relative to deep soil and parent rock, with the latter likely being the dominant factor. Zinc isotopic fractionation occurs within the plants, with root-to-shoot translocation favouring light isotope enrichment in wood and mature leaves relative to roots. In the aerial parts of this *Dichapetalum*, Zn redistribution *via* the phloem leads to heavy Zn isotope enrichment in phloem sources relative to phloem sinks, likely due to apoplastic retention and compartmentalization. Taken together, this study reveals the substantial role that *Dichapetalum* subspecies play in the Zn biogeochemical cycling of the local ecosystem.

## Methods

### Study locations and habitat of ***Dichapetalum gelonioides***

**Fig. 4:**
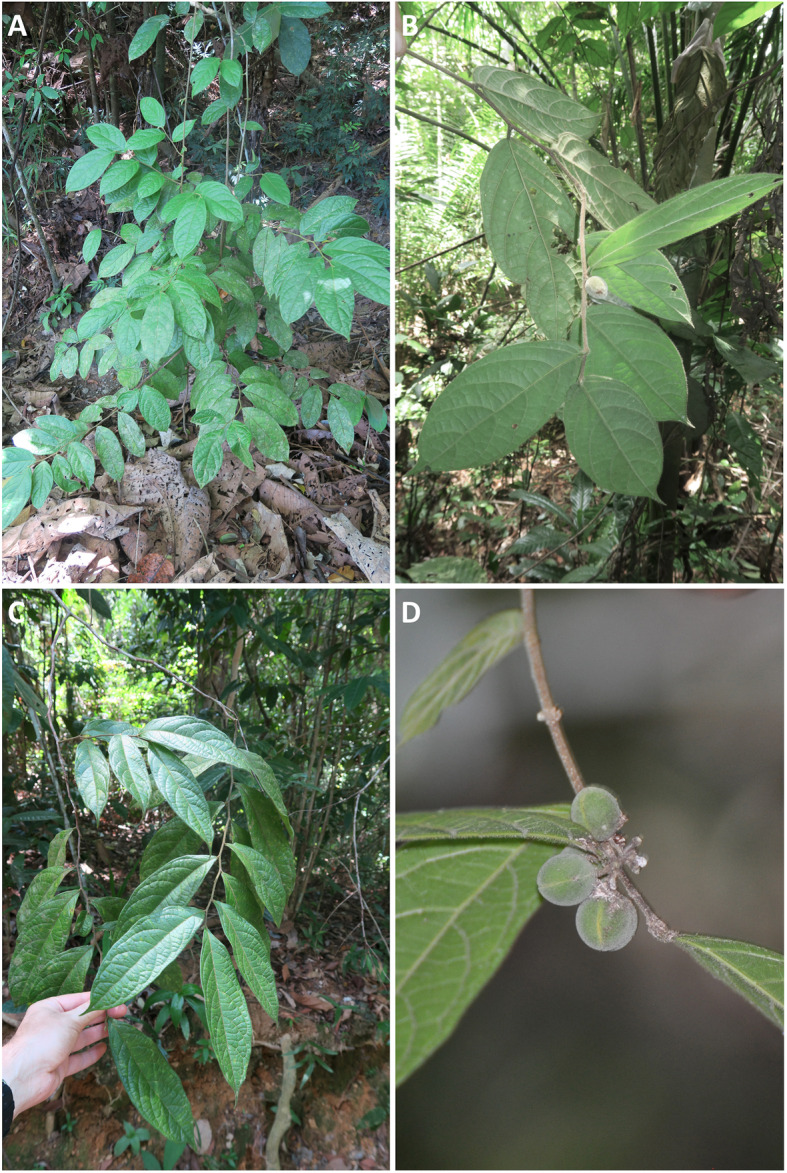
**(A)** Whole plant and **(C)** branch of *Dichapetalum gelonioides* subsp. *sumatranum*, and (**B, D**) branches of *D. gelonioides* subsp. *pilosum* with fruits in their native habitats in the Malaysian Borneo. Around the plants (**A**) there is substantial leaf litter on the topsoil

We collected the soil and plant samples in the rainforest habitat in Malaysian Borneo at the Sepilok-Kabili Forest Reserve for *D. gelonioides* subsp. *sumatranum* and at the Kebun China Forest Reserve for *D. gelonioides* subsp. *pilosum* (Fig. 4). The Sabah Forest Department granted access to the Sepilok-Kabili and the Kebun China Forest Reserves and the Sabah Biodiversity Council granted research permits. Species will further be annotated in the manuscript as *D.* subsp. *sumatranum* and *D.* subsp. *pilosum*, respectively. The Sepilok-Kabili Forest Reserve is approximately 5529 ha. The geology of the area is mudstone, sandstone and some siltstone of the Upper Miocene. The primary rain forest is dominated by *Parashorea malaanonan*-*Eusideroxylon zwageri*, *Shorea multiflora, Dipterocarpus acutangulus* and *Shorea beccariana*. This hill forest on sandstone-derived soils has a 34–40 m tall canopy. The Kebun China Forest Reserve is 149 ha and its geology also comprise of mudstone and sandstone, but this secondary forest is degraded (it has been previously logged and is partly burned).

### Collection of ***D.*** subsp. ***sumatranum*** and ***D.*** subsp. ***pilosum*** plant tissues

We collected plant tissue samples (roots, wood, old twigs, young twigs, mature (fully expanded) leaves, senescent leaves, young leaves and phloem tissue) from four individual plants of *D.* subsp. *sumatranum* and *D.* subsp. *pilosum* in the habitat following methods described by van der Ent and Mulligan (2015). The plant tissues were thoroughly rinsed with distilled water to remove surface contamination as much as possible, and then oven-dried at 70 °C for 5 days. All samples were packed for transport to Australia and gamma irradiated at Steritech Pty. Ltd. in Brisbane following Australian quarantine regulations. The plant samples were again oven-dried at 70 °C for five days to a constant mass. Each sample was ground to a fine powder, weighed and a 300 mg quantity digested using 7 mL concentrated nitric acid (HNO_3_) in an open vessel microwave oven (Milestone Start D) for 45 min at 125^o^C (~ 250 W). Following digestion, the samples were cooled and brought to volume (40 mL) with ultrapure deionised water.

### Collection of field rhizosphere soil and bedrock samples at***D.***subsp.***sumatranum***and***D.***subsp.***pilosum***field sites

We collected rhizosphere soil samples from the four individual plants of *D.* subsp. *sumatranum* and *D.* subsp. *pilosum*. In addition, we collected soil at varying depths (0–2 cm (O), 2–10 cm (A)) and bedrock samples (at > 30 cm depth) from near the roots of *D.* subsp. *pilosum*. These soils and bedrock samples were packed for transport to Australia and gamma irradiated at Steritech Pty. Ltd. in Brisbane as required by Australian quarantine regulations. The soil was air dried and sieved to < 2-mm before grinding into a fine powder using a ball mill (Retsch PM200). Similarly, the bedrock samples were crushed and ground into a fine powder. Soil and bedrock sub-samples (~ 300 mg) were strong acid leached/extracted using 9 mL concentrated HNO_3_ and 3 mL concentrated hydrochloric acid (HCl) in an open vessel microwave oven (Milestone Start D) for 1.5 h at 125^o^C (~ 250 W). Following digestion, samples were brought to volume (40 mL) with ultrapure deionised water. Soil pH was determined in a 1:2.5 soil:water (m/v) mixture after 2 h shaking and 1 h rest. Exchangeable trace elements were extracted in 0.01 M strontium nitrate (Sr(NO_3_)_2_) at a soil: solution ratio of 1:4 (m/v) (10 g soil with 40 mL solution) and 2 h shaking time (adapted from Kukier and Chaney 2001). A diethylene triamine pentaacetic acid (DTPA)-extractant was used to determine the potentially phytoavailable trace concentrations in soils following the method of Becquer et al. (1995), which was adapted from the original method by Lindsay and Norvell (1978), with the following modifications: excluding triethanolamine (TEA), adjusted to pH 5.3, 5 g soil with 25 mL extractant, and extraction time of 1 h.

### Total Zn analysis of soils, bedrock and plant tissues

The plant digests and soils and bedrock digests/extracts were analysed by inductively coupled plasma - atomic emission spectroscopy (ICP-AES; Thermo Scientific iCAP 7400) for Zn concentrations. In-line internal addition standardization using yttrium was used to correct for matrix-based interferences. Quality controls included matrix blanks, Standard Reference Material (NIST 1570a Spinach Leaves digested with HNO_3_). The measured values Zn concentrations (average ± standard error: 81.5 ± 0.33 µg g^− 1^) were in close agreement with the certified value of 82.3 µg g^− 1^.

### Estimation of the uptake, translocation and bioconcentration coefficient factors

The Zn uptake (root/soil) factor (UF) is the ratio of Zn concentrations in roots to that of the rhizosphere soil. The Zn translocation (leaves/root) factor is given as the ratio of Zn concentrations in leaves to that of the roots. The Zn Bioconcentration Coefficient (BC) (leaf/soil) is the ratio of Zn concentrations in leaves to that of the rhizosphere soils.

### Zinc isotope ratio analysis

Plant tissue samples (*n* = 2) for *D.* subsp. *sumatranum* and *D.* subsp. *pilosum* were digested using a protocol based on US EPA method 3502 (US Environmental Protection Agency 1996). Approximately 0.2 g of homogenized powder was weighed into a 15-mL perfluroalkoxy (PFA) vial, then 8 mL concentrated HNO_3_ was added. The sample was cold digested overnight, followed by the addition of 1 mL hydrogen peroxide (H_2_O_2_). The solution was monitored for effervescence/spillage then a second addition of 1 mL H_2_O_2_ was performed. The resulting 10 mL solution was decanted into Teflon vessels for closed vessel microwave digestion at 180 °C for 30 min (Anton-Parr; 1000 W). After digestion, the solution was transferred to clean 15-mL PFA vials and sub-sampled for total Zn analysis by inductively coupled plasma – mass spectrometry (ICP-MS) (Agilent 8900) and column purification (resin anion-exchange chromatography) prior to multi-collector inductively coupled plasma-mass spectrometry (MC-ICP-MS) analysis. The accuracy of digestion and analysis methods for determination of Zn concentrations was monitored by analysing Certified Reference Materials (‘CRM’) 1567b (wheat flour) and 1573a (tomato leaves). The Zn concentrations found in CRMs samples were in close agreement with the certified values.

Total Zn concentrations in soil and bedrock samples (n = 2) from different depths in the profile (O, A, B and R) were determined by weighing between 0.2 and 0.3 g dry mass into a 15-mL perfluoroalkyl (PFA) vial. The sample was digested for 4 days at 120 °C on a hot plate in a mixture of concentrated HNO_3_ and concentrated hydrofluoric (HF) acids. The samples were evaporated to dryness, during which HNO_3_ was added to drive off the HF. The samples were digested for a further 4 days in HNO_3_/H_2_O_2_ mixture. After digestion, the samples were separated into two aliquots for total Zn concentrations using ICP-AES or ICP-MS and column purification (ion exchange chromatography) prior to MC-ICP-MS analysis of Zn isotope ratios. The accuracy of digestion and analysis methods for determination of Zn concentrations was monitored by analysing CRM 2709a (San Joaquin soil). The Zn concentrations found in the CRM were in close agreement with the certified value.

Zinc was separated from other matric elements (including copper (Cu), Ni, iron (Fe)) using a modified column purification procedure outlined by Sossi et al. (2014). Briefly, aliquots of plant or soil digest solutions (1000–1200 ng Zn) were evaporated in PFA vials to dryness at 80 °C. The samples were converted to the chloride form by adding 2 mL of 6 M HCl and evaporating to dryness. The samples were taken up in 1 mL of 6 M HCl for loading into Poly-Prep™ columns loaded with 2 mL of AG1-X8 100–200 mesh ion-exchange resin. The column cleaning and elution procedures are summarized in Supplementary Table 2. 6 M HCl was used to elute major elements such as aluminium (Al), sodium (Na), calcium (Ca) and doubly charged elements (titanium (Ti) and barium (Ba)) from the column. Transition metals such as Cu and Fe were then eluted with 0.5 M HCl, and Zn fraction was then eluted with 15 mL of 0.5 M HNO_3_. The collected Zn fraction was evaporated to dryness at 90 °C and taken up in 3 mL of 2 % v/v HNO_3_ for MC-ICP-MS analysis. An aliquot of 1 mL was sub-sampled to measure total Zn concentrations and range of matrix elements (e.g. Cu, Ni, Fe, Ca, Na, potassium (K), Ti) by ICP-MS (Agilent 8900) to check Zn recovery and that matrix and interfering elements had been removed from the Zn fraction. Recoveries of Zn from sample columns were between 96 and 136 %.

Zinc isotope ratios were determined using MC-ICP-MS (Neptune, Thermo Scientific) housed at Waite Campus, Adelaide, Australia. The samples were measured in 2 % HNO_3_ at Zn concentrations between 350 and 400 µg L^− 1^. Samples were measured in low resolution mode, using H cones and a 50 µL min^− 1^ nebulizer attached to a Scott double pass quartz spray chamber. Masses ^62^Ni, ^63^Cu, ^64^Zn, ^65^Cu, ^66^Zn, ^67^Zn and ^68^Zn were measured simultaneously, with ^62^Ni monitored to correct for ^64^Ni interference on ^64^Zn (no interference on ^64^Zn due to ^64^Ni was observed in this study). A single run consisted of 1 block of 35 cycles with 4 s integration time. A baseline and peak centre were performed before each run. The standard-sample-standard bracketing method was used using Zn isotopic standard IRMM 3702 as the bracketing standard. Samples were spiked with 400 µg L^− 1^ Cu for instrumental mass bias correction by external normalization using the exponential law (Marechal et al. 1999). An in-house isotopic Cu standard (‘STD1’) with a copper isotope ratio, ^65^Cu/^63^Cu = 0.45 (similar to that of NIST 976 = 0.45) was used for instrumental mass bias correction. An on-peak zero correction was performed by measuring a blank before and after each sample and bracketing standard. The Zn isotope results are reported in ‘per mil’ notation with δ^66^Zn defined as:

δ^66^Zn = (^66^Zn/^64^Zn_sample_/^66^Zn/^64^Zn_IRMM 3702_ -1) x 1000

Zinc isotopes have previously been reported in the literature relative to the JMC-Lyon reference value. The conversion factor between the two standards can be determined using the following formulae (Sossi et al. 2014):

δ^66^Zn_3702_ = δ^66^Zn_JMC_ + 0.30

The analytical blank (digestion and column) represented < 3 % of the total Zn concentration in samples for MC-ICP-MS analysis. The accuracy of digestion, purification and analysis procedures for the determination of δ^66^Zn in plant tissues, soils and bedrock samples was assessed using the CRM’s NIST1567b (wheat flour) and NIST1573a (tomato leaves); and CRM NIST2709a (San Joaquin soil). The measured values (average ± 2 SD; CRM 1567b δ^66^Zn = 0.98 ± 0.04; CRM 1573a δ^66^Zn = 0.45 ± 0.06; CRM 2709a δ^66^Zn = -0.02 ± 0.06) were in close agreement with published values (CRM 1567b δ^66^Zn = 0.95 ± 0.05; CRM 1573a δ^66^Zn = 0.49 ± 0.09; CRM 2709a δ^66^Zn = -0.02) (Araujo et al. 2017; Wiggenhauser et al. 2018).

## Supplementary Information

**Additional file 1:** **Supplementary Table S1**. Soil pH, strontium nitrate- and diethylene triamine pentaacetic acid (DTPA)-extractable Zn concentrations (μg g^-1^), total Zn concentrations and δ^66^Zn isotope ratios (‰) of soil profile (0–2cm (O), Soil 2–10cm (A)) and bedrock (30 cm (R)) samples collected from the native habitat of Dichapetalum gelonioides subsp. pilosum. SD = standard deviation (instrumental analysis variability). **Supplementary Table S2**. Procedure for separating and purifying Zn for isotope measurements by multi-collector inductively coupled plasma-mass spectrometry (MC-ICP-MS).

## Data Availability

The datasets used and/or analysed during the current study are available from the corresponding author on reasonable request.

## Abbreviations

BC: Bioconcentration Coefficient
CRM: Certified Reference Materials
DTPA: Diethylene triamine pentaacetic acid
HMAs: Heavy metal ATPases
ICP-AES: Inductively-coupled plasma - atomic emission spectroscopy
ICP-MS: Inductively-coupled plasma–mass spectrometry
IRMM: Institute for Reference Materials and Measurements
JMC: Johnson Matthey Company
m/v: Mass-volume ratio
MC-ICP-MS: Multi-collector inductively coupled plasma-mass spectrometry
n: Number of samples
NIST: National Institute of Standards and Technology
PFA: Perfluroalkoxy
SD: Standard deviation
STD1: In-house isotopic copper standard
TEA: Triethanolamine
UF: Uptake factor
v/v: Volume-volume ratio
δ^66^Zn: Zinc isotope ratio
