# Supplementary material for: Isotopic signatures reveal zinc cycling in the natural habitat of hyperaccumulator Dichapetalum gelonioides subspecies from Malaysian Borneo
Source: BMC Plant Biol. 2021 Sep 27;21:437. doi: 10.1186/s12870-021-03190-4 (PMC8474765; doi:10.1186/s12870-021-03190-4)
Supplement: Supplementary file 1 — Additional file 1: Supplementary Table S1. Soil pH, strontium nitrate- and diethylene triamine pentaacetic acid (DTPA)-extractable Zn concentrations (μg g-1), total Zn concentrations and δ66Zn isotope ratios (‰) of soil profile (0–2cm (O), Soil 2–10cm (A)) and bedrock (30 cm (R)) samples collected from the native habitat of Dichapetalum gelonioides subsp. pilosum. SD = standard deviation (instrumental analysis variability). Supplementary Table S2. Procedure for separating and purifying Zn for isotope measurements by multi-collector inductively coupled plasma-mass spectrometry (MC-ICP-MS). [file 12870_2021_3190_MOESM1_ESM.docx]

**Supplementary Tables 1–2**

**Authors:** Antony van der Ent, Philip Nti Nkrumah, Mark G. M. Aarts, Alan J. M. Baker, Fien Degryse, Chris Wawryk, Jason K. Kirby

**Title:** Isotopic signatures reveal zinc cycling in the natural habitat of hyperaccumulator *Dichapetalum* *gelonioides* subspecies from Malaysian Borneo

**Supplementary Table 1.** Soil pH, strontium nitrate- and diethylene triamine pentaacetic acid (DTPA)-extractable Zn concentrations (µg g^-1^), total Zn concentrations and δ^66^Zn isotope ratios for soils and bedrock samples collected from the native habitat of *Dichapetalum gelonioides* subsp. *pilosum*.

| **Sample type** | **pH** | **Sr(NO_3_)_2_-Zn**  **(µg g^-1^)** | **DTPA-Zn**  **(****µg g^-1^)** | **Total Zn  (µg g^-1^)** | **δ^66^Zn (‰)** | **SD** |
| --- | --- | --- | --- | --- | --- | --- |
| Soil 0–2 cm (O) | 6.64 | 0.65 | 30 | 85 | 0.13 | 0.06 |
| Soil 2–10 cm (A) | 6.18 | 0.35 | 5.5 | 40 | -0.02 | 0.06 |
| Soil 10–30 cm (B) | 4.95 | 1.00 | 0.77 | 25 | -0.15 | 0.08 |
| Bedrock 30 cm (R) | 5.68 | 0.08 | 0.27 | 45 | -0.90 | 0.08 |

SD = standard deviation (instrumental analysis variability)

**Supplementary Table 2.** Procedure for separating and purifying Zn for isotope measurements by MC-ICP-MS.

| Step | **Process details** |
| --- | --- |
| Clean Resin | 6 ml 0.5M HNO_3_ |
|  | Rinse with 2 reservoirs MQ H_2_O |
|  | 6 ml 3M HCl |
|  | 6 ml H_2_O |
|  | Equilibrate with 4 mL 6M HCl |
| Load Sample | Load sample in ~1 mL 6M HCl |
|  | Rinse beaker with 1ml 0.5M HNO_3_ |
| Elute matrix and base metals | 30 mL 6M HCl |
|  | 10 ml 0.5M HCl |
|  | Wash column with 1ml 0.5M HNO_3_ |
| **Elute Zn** | 15ml 0.5M HNO_3_ |
|  | Evaporate to dryness |
|  | Take up in 2% HNO_3_ for measurement on MC-ICP-MS |
